# High-speed electro-optic modulation in topological interface states of a one-dimensional lattice

**DOI:** 10.1038/s41377-023-01251-x

**Published:** 2023-08-29

**Authors:** Yong Zhang, Jian Shen, Jingchi Li, Hongwei Wang, Chenglong Feng, Lei Zhang, Lu Sun, Jian Xu, Ming Liu, Ying Wang, Yonghui Tian, Jianwen Dong, Yikai Su

**Affiliations:** 1 State Key Laboratory of Advanced Optical Communication Systems and Networks, Department of Electronic Engineering, Shanghai Jiao Tong University, Shanghai, 200240 China; 2 Center for Advanced Electronic Materials and Devices, Shanghai Jiao Tong University, Shanghai, 200240 China; 3 Institute of Microelectronics and Key Laboratory for Magnetism and Magnetic Materials of MOE, School of Physical Science and Technology, Lanzhou University, Lanzhou, 730000 Gansu China; 4 State Key Laboratory of Optoelectronic Materials and Technologies & School of Physics, Sun Yat-sen University, Guangzhou, 510275 China

**Keywords:** Integrated optics, Photonic crystals

## Abstract

Electro-optic modulators are key components in data communication, microwave photonics, and quantum photonics. Modulation bandwidth, energy efficiency, and device dimension are crucial metrics of modulators. Here, we provide an important direction for the miniaturization of electro-optic modulators by reporting on ultracompact topological modulators. A topological interface state in a one-dimensional lattice is implemented on a thin-film lithium-niobate integrated platform. Due to the strong optical confinement of the interface state and the peaking enhancement of the electro-optic response, a topological cavity with a size of 1.6 × 140 μm^2^ enables a large modulation bandwidth of 104 GHz. The first topological modulator exhibits the most compact device size compared to reported LN modulators with bandwidths above 28 GHz, to the best of our knowledge. 100 Gb/s non-return-to-zero and 100 Gb/s four-level pulse amplitude modulation signals are generated. The switching energy is 5.4 fJ/bit, owing to the small electro-optic mode volume and low capacitance. The topological modulator accelerates the response time of topological photonic devices from the microsecond order to the picosecond order and provides an essential foundation for the implementation of large-scale lithium-niobate photonic integrated circuits.

## Introduction

Lithium-niobate-on-insulator (LNOI) has emerged as an important platform for integrated photonics due to its excellent properties, such as the strong electro-optic effect, large nonlinear coefficient, and wide transparency window^1–3^. High-performance electro-optic modulators have been demonstrated using the Pockels effect on the thin-film LN integrated platform^4–6^ and are at the heart of ultralarge-capacity optical communication, terahertz wireless communication, microwave signal processing, sensing, and quantum technology^7^. The Mach‒Zehnder interferometer (MZI) or ring-assisted MZI modulators on thin-film LN have the advantages of a large bandwidth and low driving voltage^1,4,8,9^. However, they require long phase shifters with lengths of 3~20 mm, resulting in a large device footprint. LN microring or racetrack modulators can support large bandwidths^10,11^, but the bending radius and device size are still large and limited by the anisotropy of the LN thin film. Recently, substantial progress has been made in LN modulators based on Fabry-Perot (FP) cavities^12^ and photonic crystal nanobeams^5^. Unfortunately, the length of the FP cavity-based modulator is as long as 500 μm, while the bandwidth of the nanobeam-based modulator is limited to 17.5 GHz. Next-generation electro-optic modulators require high-density integration, a large bandwidth, and low power consumption^13^, especially for applications where space and power consumption are constrained such as co-packaged optics and optical I/O^14^, which are challenging to achieve with established integrated devices.

Topological phase transition is an essential component in various physical systems for diverse applications, including condensed matter^15^, cold atoms^16^, acoustics^17^, and mechanics^18^. Recently, topological photonics has attracted remarkable attention due to its unique properties, such as robust transport of light and immunity to defects or disorders^19,20^. Topological photonics can be applied in the structures of one-dimensional (1D), 2D, and 3D topological photonic crystals^21,22^, coupled microring resonators^23^, metamaterials^24^, and quasicrystals^25^. Various topological photonic devices have been demonstrated, such as cavities^26^, filters^27^, splitters^28^, vortex generators^29^, nonlinear and quantum devices^30,31^, and active devices for lasing^32^ and switching^33,34^. Due to the tight optical confinement and robust transport of light in integrated topological structures, integrated LN waveguides with 1D topological interface states can help address the challenges of next-generation electro-optic modulators. The 1D topological cavity features a shorter length than the conventional 1D Bragg grating structures. Unlike the photonic crystal nanobeam cavity with multiple resonant modes, the topological cavity allows flexible control of the Q factor and mode volume while strictly maintaining single-mode operation and avoiding the mode number control.

In this work, we report high-speed and energy-efficient electro-optic modulation in topological interface states of a 1D microstructure lattice on a silicon-nitride-loaded LNOI platform. A topological interface state is formed between two topological photonic crystals with distinct topological invariants and surface impedance in the 1D lattice based on the classic Su-Schrieffer-Heeger (SSH) model. The interface state enables the first topological modulator with a compact size of only 1.6 × 140 μm^2^, which is the most compact thin-film LN modulator with a bandwidth exceeding 28 GHz. Low radio frequency (RF) loss and small capacitance are achieved due to the small electro-optic modal volume and short electrode length, yielding ultralow energy consumption of 5.4 fJ/bit. Peaking enhancement in the electro-optic response of the topological cavity is utilized to break the photon lifetime-limited bandwidth, resulting in a large bandwidth of 104 GHz. As an application example, the topological modulator is operated with a non-return-to-zero (NRZ) signal of up to 100 Gbaud. Our topological modulator shows excellent performance in terms of ultrasmall size, high speed, and energy efficiency; our study accelerates the response time of topological photonic devices from the microsecond order to the picosecond order and promotes applications of topological devices in optical communications, microwave photonics, and quantum information processing.

## Results

### Design of the topological interface state on the LN platform

**Fig. 1:**
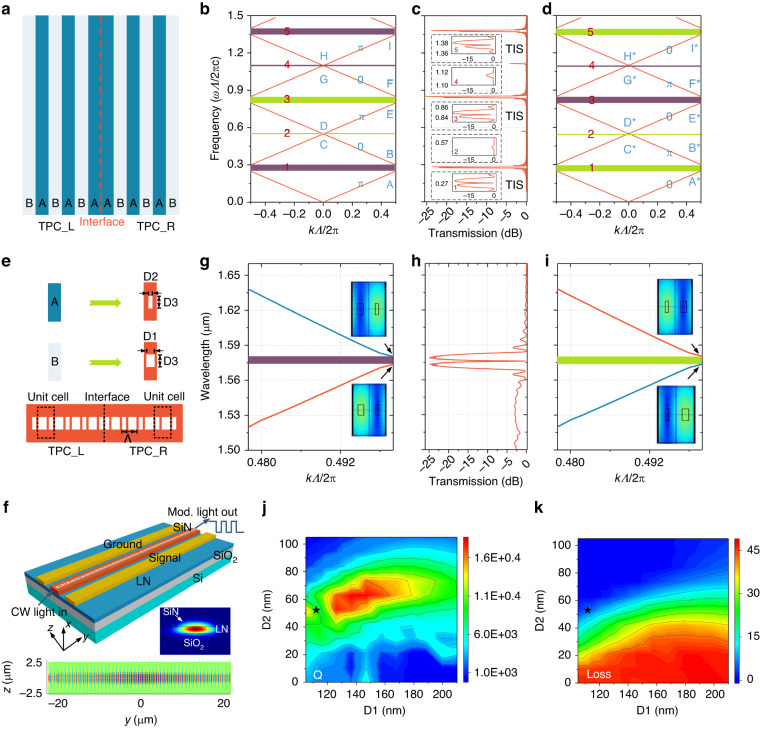
Topological photonic crystal and its bulk band diagram on silicon-nitride-loaded LN platform. **a** 1D TPC cavity based on a dielectric AB layered structure. Band structures of the **b** left and **d** right TPCs. The Zak phase of each isolated band is marked with blue letters. The gap topological invariants of each gap are labeled purple when sgn[*ζ*] > 0 and light green when sgn[*ζ*] < 0. **c** Simulated transmission spectrum of the 1D TPC cavity consisting of the two inverted TPC structures. Resonance peaks are located in the center of the 1st, 3rd, and 5th stopbands, which correspond to the topological interface states (TIS). The smaller panels are zoom-in views of the simulated transmission spectra of stopbands 1~5, to provide a clearer illustration of the presence or absence of topological boundary states. **e** Schematic of the rectangular air holes on an integrated waveguide. Periodic rectangular holes are utilized to tailor the effective index of integrated waveguides and replace the dielectric AB layered structures. **f** 3D view of the modulator based on the topological interface state. Insets show the simulated optical field distribution of the hybrid Si_3_N_4_-LNOI waveguide and the topological boundary state. The simulated optical field distributions of the Si_3_N_4_-LN waveguide and the topological boundary state are monitored at the Thru port of the topological structure and the center of the LN thin film, respectively. Band structures of the **g** left and **i** right integrated TPC based on periodic rectangular holes. **h** Simulated transmission spectrum of the integrated TPC cavity. Calculated (**j**) loaded Q factors and **k** insertion losses of the topological edge mode for different D1 and D2 values. CW continuous wave, Mod. modulated, TPC_L left TPC, TPC_R right TPC

We begin with a dielectric AB layered structure as shown in Fig. 1a. The 1D topological cavity consists of a left topological photonic crystal (TPC) and a right TPC. To obtain a topological interface state at one photonic bandgap, the gap topological invariants on the left and right TPCs are required to ensure a difference of *π*. A method of crossing a topological transition point is used to change the sign of the topological invariant. The left TPC is formed by periodically stacked dielectrics A and B. The relative permittivity *ε* and permeability *μ* of layers A and B are *ε*_a_ = 3.39, *ε*_b_ = 3.24, *μ*_a_ = *μ*_b_ = 1. The thicknesses of layers A and B are *d*_a_ = *d*_b_ = *Λ*/2, where *Λ* is the period of the TPC. The right TPC is mirror symmetric with the left TPC, which consists of the inverted unit cells. Since the *n*_a_*d*_a_ + *n*_b_*d*_b_ of the left and right TPCs are the same, the positions of the energy band are not altered during the inversion process. The calculated band structures of the left and right TPCs are depicted in Fig. 1b, d, respectively. For the *j*th energy band of the TPC, the Zak phase is defined as follows^35,36^:

1 $${\theta }_{j}^{Zak}={\int }_{-\pi /\varLambda }^{\pi /\varLambda }\left[i\int dy\cdot \varepsilon (y){u}_{j,k}^{\ast }(y){\partial }_{k}{u}_{j,k}(y)\right]dk$$

where *u*_*j,k*_(*y*) is the periodic-in-cell part of the Bloch electric field eigenfunction of a state on the *j*th band with wave vector *k*. *u*_*j,k*_(*y*) can be calculated using the transfer-matrix method for a binary TPC^37^. The dielectric layered TPC structure has two inversion centers, and the center of layer A is selected as the origin of our study. The Zak phase of the *j*th band is either 0 or *π* for the perfect binary TPC. The Zak phase of each isolated band is calculated using Eq. (1) and marked with blue letters in Fig. 1b, d. The Zak phases of each isolated band can also be obtained by analyzing the symmetry properties of the interface states at the two symmetry points in the Brillouin region (see Supplementary Note S1).

The gap topological invariants of the *n*th gap, i.e., the sum of Zak phases of all the isolated bands below the *n*th gap, can be used to predict the existence of an interface state in a bandgap. The gap topological invariants of the *n*th gap can be obtained from the following relationship^37^:

2 $$\mathrm{sgn}[{\zeta }^{(n)}]={(-1)}^{n}{(-1)}^{l}\exp \left(i\mathop{\sum }\limits_{j=0}^{n-1}{\theta }_{j}^{Zak}\right)$$

where the integer *n* is the number of the gaps, and *l* is the number of crossing points under the *n*th gap. Using Eq. (2), the gap topological invariants of each gap for the left and right TPCs are obtained and labeled by purple when sgn[*ζ*] > 0 and light green when sgn[*ζ*] < 0. The 1st, 3rd, and 5th gaps of the TPCs exhibit different gap topological invariants, indicating that a topological phase transition occurs when the lower and upper edges of the gap cross each other. The topological interface state exists in these photonic gaps when combining the left and right TPCs together. We use the finite-difference time-domain (FDTD) method to simulate the transmission spectrum of the 1D topological cavity consisting of the two inverted TPC structures, as shown in Fig. 1c. Resonance peaks are located in the center of the 1st, 3rd, and 5th stopbands, which correspond to the topological interface states. The 2nd and 4th gaps of the two TPCs have the same gap topological invariants, thus, no resonance peak is observed in these two stopbands. The prediction of the existence of boundary states based on topological invariants coincides with the transmission spectrum simulated by the FDTD method. There are some small fluctuations at the edges of the band gaps, which are caused by the slow light phenomenon due to the sharp change in group refractive index at the band edges. Compared to the high extinction ratio (>20 dB) of the topological edge states in the center of the band gaps, the extinction ratio of these fluctuations at the edges of the band gaps is very small, and we believe that they will not have any impact on the topological edge states.

Next, we consider the transformation of topological photonic crystals with the abovementioned layered structures into an integrated topological cavity on a thin-film LN platform. Si_3_N_4_-loaded LNOI integrated waveguide is used in the design to avoid directly etching the LN thin film and offers a promising direction to achieve large-scale integration of passive and active LNOI devices. The calculated optical confinement factor in the thin-film LN is 61.5%, which can take advantage of the strong electro-optic effect of the thin-film LN. The cross-section and simulated optical field distribution of the hybrid Si_3_N_4_-LNOI waveguide are shown in the insets of Fig. 1f. For the on-chip design, periodic rectangular air holes are utilized to tailor the effective index of integrated waveguides and replace the dielectric AB layered structures in the above TPC cavity, as depicted in Fig. 1e. The equivalent refractive index of the waveguides with subwavelength-scale rectangular air holes is calculated using effective-medium theory^38^ (see Supplementary Note S2). The integrated left TPC is composed of periodical unit cells, which contain two rectangular air holes with different sizes on the Si_3_N_4_ layer. The right TPC is mirror symmetric with the left TPC, which consists of inverted unit cells. The two TPCs exhibit a common photonic band structure but are distinct in terms of the gap topological invariant, as shown in Fig. 1g, i. By constructing an interface between the left and right TPCs, a topological interface state can be generated inside the photonic gap, which is verified by the simulated transmission spectrum of the integrated TPC cavity on the Si_3_N_4_-loaded LNOI platform (Fig. 1h). The topological phase transition can also be confirmed by the changes in the symmetries of the interface states. The amplitude of the wave function of the band-edge state at the origin (the center of the air hole with a smaller size) is either zero or the maximum, as shown in the insets of Fig. 1g, i. The wave functions exhibit different parities: (1) the left TPC supports an anti-symmetric state with a zero magnitude at the origin for the lower edge of the gap and a symmetric state with the maximum magnitude at the origin for the upper edge of the gap, and (2) the right TPC supports a symmetric state with the maximum magnitude at the origin for the lower edge of the gap and an anti-symmetric state with a zero magnitude at the origin for the upper edge of the gap. A topological edge state can be predicted to exist inside the gap if the states at the lower edge of the common gap of the left and right TPCs belong to different types. The parameters of the integrated TPC cavity are as follows: the waveguide width is 1600 nm, the period Λ of the unit cells is 420 nm, the number of unit cells is 170, and the D1, D2, and D3 of the rectangular air holes are 150 nm, 80 nm, and 674 nm, respectively.

The Q factor, mode volume, insertion loss, and extinction ratio are important metrics for electro-optic modulators to evaluate the LN-based TPC integrated cavity. We calculate the loaded Q factors and insertion losses of the topological edge modes for different D1 and D2 values (Fig. 1j, k). The topological nanocavity shows a maximum loaded Q factor of 18,400 with a mode volume of 1.98 μm^3^. This *Q* value is well suited to the requirements of high-speed electro-optic modulators since a higher Q value results in a long photon lifetime inside the cavity, limiting the electro-optic modulation bandwidth. The Q factor can be manipulated by changing the dimensions of the air holes and the number of unit cells. Unlike the conventional photonic crystal nanobeam cavity with multiple resonant modes^39^, the topology-based cavity enables the flexible control of the Q factor and mode volume while strictly maintaining single mode operation and avoiding the mode number control. The insertion loss of the topological edge mode can be ignored when the D2 value increases beyond 60 nm. The simulated extinction ratio of the topological cavity can reach 33.8 dB (see Supplementary Note S3). The electric field distribution of the topological interface state of the TPC cavity is plotted in the insets of Fig. 1f. We analyze the mode field of the Zak phase of the topological interface states in the classical SSH model using the tight-binding approximation (see Supplementary Note S4).

### Device fabrication and static characterization

The 3D schematic of the proposed modulator based on the topological cavity is depicted in Fig. 1f. An X-cut LNOI wafer with a 300-nm-thick LN thin film is adopted in this work. The topological cavity is oriented along the y-axis to take advantage of the highest electro-optic coefficient *r*_33_ of the LN thin film. To maximize the in-plane electric field *E*_z_, we apply an electric field through coplanar microelectrodes beside the TPC cavity. The simulated static electric field distribution and electro-optic overlap integral Γ of the proposed topological structure can be found in Supplementary Note S5. The calculated capacitance of the microelectrodes is 36 fF/mm. The topological modulator with an electrode length of 150 μm exhibits a small capacitance C of only 5.4 fF, which helps to achieve higher energy efficiency (see Supplementary Note S5).

**Fig. 2:**
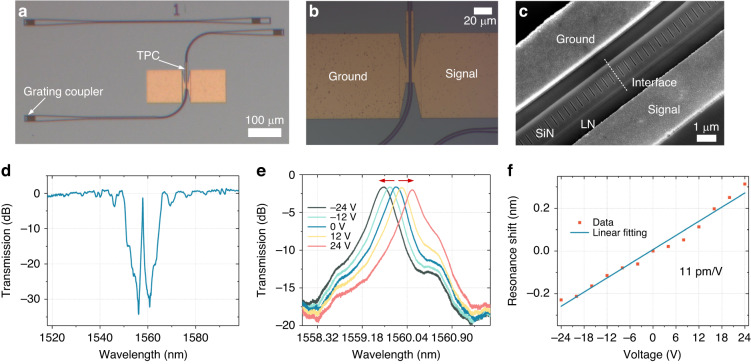
Fabrication and static characterization of a topological Pockels modulator. **a** Optical microscope graph of the fabricated topological modulator with grating couplers. **b** Magnified photo of the device with gold electrodes. **c** Scanning electron microscope (SEM) image of the topological structure. **d** Measured transmission spectrum of the TPC cavity without gold electrodes. **e** Measured transmission spectra of the fabricated TPC cavity upon applying different voltages. **f** Electro-optic tuning of the boundary states versus applied voltages

Figure 2a–c show optical microscope and scanning electron microscopy (SEM) images of the fabricated topological modulator device (see “Methods” for the details of device fabrication). Grating couplers are used to couple the light into and out of the topological chip. The measured transmission spectrum of the fabricated topological cavity without microelectrodes is plotted in Fig. 2d. In agreement with the simulation results, only one peak appears in the center of the photonic bandgap, corresponding to the boundary state of the topological cavity. The 3-dB bandwidth, quality factor Q, insertion loss, and extinction ratio are 0.172 nm, 9066, 1.3 dB, and 32 dB, respectively.

To demonstrate electro-optic tuning, a topological cavity with gold microelectrodes is fabricated and characterized. Figure 2e illustrates the electro-optic tuning of the topological boundary state. Upon applying an electric field across the topological cavity, the resonance wavelengths shift, indicating a change in the effective index of the topological cavity. No degradation of the spectrum shape occurs, even when the voltage increases to 24 V. A clear linear dependence of the wavelength shift on the applied voltage is obtained with a linear tuning efficiency of Δλ/ΔV = 11 pm/V (Fig. 2f). The high tuning efficiency is attributed to the large electro-optic overlap and the strong optical field confinement in the small topological cavity.

### Modulation bandwidth

**Fig. 3:**
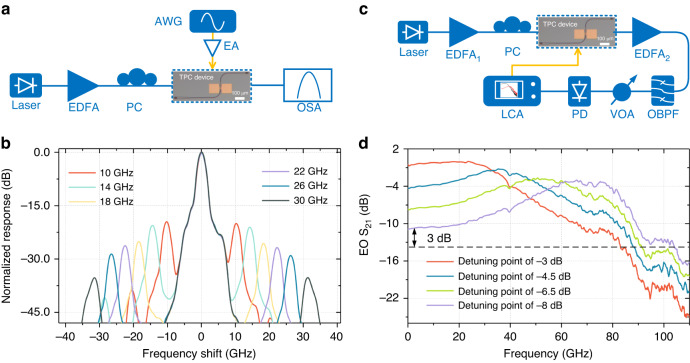
Modulator bandwidth and electro-optic characterization. **a** Measurement setup for sideband testing. **b** Optical spectra at the output of the topological modulator for various input RF frequencies. The peak located in the center of the spectra is the input optical carrier, while the two peaks on both sides correspond to the generated modulation sidebands. **c** Experimental setup for measuring the electro-optic bandwidth of the topological modulator. **d** Measured electro-optic S_21_ responses with different wavelength detuning Δλ. EDFA erbium-doped fiber amplifier, PC polarization controller, EA electrical amplifier, OBPF optical bandpass filter, VOA variable optical attenuator, PD photodetector, EO electro-optic

We test the sidebands generated by the modulation of RF signals at different frequencies, to investigate the high-frequency response of the topological modulator. Figure 3a shows the measurement setup for sideband testing (see “Methods” for the details of the setup). Modulation responses for the topological device are depicted in Fig. 3b. The peak located in the center of the spectra is the input optical carrier, while the two peaks on both sides correspond to the generated modulation sidebands. The sidebands show an offset equal to the applied modulation frequency, relative to the input optical carrier. As the modulation frequency increases, the power of the measured sideband decreases. The modulation efficiency is significantly higher than the previously reported results of the LN racetrack modulator^40^ and silicon carbide microring modulator^41^ and contributes to a better microelectrode structure and large Pockels coefficient.

The modulation bandwidth for a cavity modulator is mainly limited by the photon lifetime *τ* in the cavity, resulting in a first-order filter with a bandwidth of 1/2*πτ*, and the RC time constant of the phase shifter, leading to another first-order filter^42^. Attributed to the short electrode length and small capacitance of the lumped-element topological modulator, the RC-limited bandwidth is beyond 1.1 THz (see Supplementary Note S6). Therefore, the bandwidth of the topological modulator is mainly limited by the photon lifetime. To show a higher modulation bandwidth, we select another topological modulator device on the same chip with a lower Q factor of 5400. The photon lifetime is calculated to be *τ* = Q*λ*/2*πc* = 4.5 ps. Thus, the modulation bandwidth for this modulator is limited to 35 GHz. Peaking arising from the intrinsic dynamics in the optical domain has been utilized to extend the modulation bandwidth of silicon microring modulators^43,44^. Here, we show that the transient peaking response can also be used to extend the operating frequency range of the LN topological modulator beyond the cutoff limited by the photon lifetime. The small-signal electro-optic S_21_ responses of the fabricated topological modulator are characterized by a 110-GHz lightwave component analyzer (LCA). The measurement setup is shown in Fig. 3c (see “Methods” for the details of the setup). The measured S_21_ responses are plotted in Fig. 3d for the four laser wavelength detuning points corresponding to 3, 4.5, 6.5, and 8 dB down from the on-resonance transmission maximum power level. The modulation response is highly dependent on the wavelength detuning. The measured 3-dB electro-optic bandwidth of the topological modulator is 37, 67, 87, and 104 GHz at wavelength detuning points of −3, −4.5, −6.5, and −8 dB, respectively. The overshoot in the electro-optic S_21_ response is the consequence of the interference effect between the resonant light inside the topological cavity and the input light from the waveguide. The frequency shift between the input light and the resonance acts as a source term, thus, additional phase retardation of *π*/2 accumulates, and the interference conditions between the boundary state and the waveguide change from destructive interference to constructive interference, leading to a peak in the electro-optic response. The modulation frequency of the peak in the S_21_ response roughly corresponds to the detuning between the input light frequency and the resonant frequency. This improvement in bandwidth is achieved at the cost of reduced modulation efficiency, but it offers a clear route to reconfigure the topological modulator.

A small-signal model based on perturbation theory can be used to analyze the peaking enhancement in the electro-optic response^42^. The small-signal S_21_ response of the topological cavity modulation can be derived as follows:

3 $${S}_{21,EO}=\sqrt{\frac{2}{\tau }}\mathrm{Re}\left(\left[\frac{\frac{1}{\tau }\bar{a}{(-i\bar{a}\overline{\delta {\omega }_{r}})}^{\ast }}{i{\omega }_{r}-i{\omega }_{0}+i{\omega }_{m}+\frac{2}{\tau }+\frac{1}{{\tau }_{r}}}+\frac{-i\frac{1}{\tau }\bar{a}\overline{\delta {\omega }_{r}}{(\bar{a})}^{\ast }}{i{\omega }_{r}-i{\omega }_{0}-i{\omega }_{m}+\frac{2}{\tau }+\frac{1}{{\tau }_{r}}}\right]{e}^{-i{\omega }_{m}t}\right)$$

where *δω*_*r*_ is the resonant angular frequency change when applying a small voltage, *a* is the optical field traveling inside the topological cavity, *τ*_*r*_ is the radiation coupling between the topological cavity and the cladding, and *ω*_*m*_, *ω*_0_, and *ω*_*r*_ represents the modulation frequency, the input light frequency, and the resonance frequency, respectively. The detailed perturbative derivation of Eq. (3) and the simulated small-signal response of the topological cavity modulation can be found in Supplementary Note S7. The simulated bandwidth closely matches the experimentally tested bandwidth.

### Data modulation testing

**Fig. 4:**
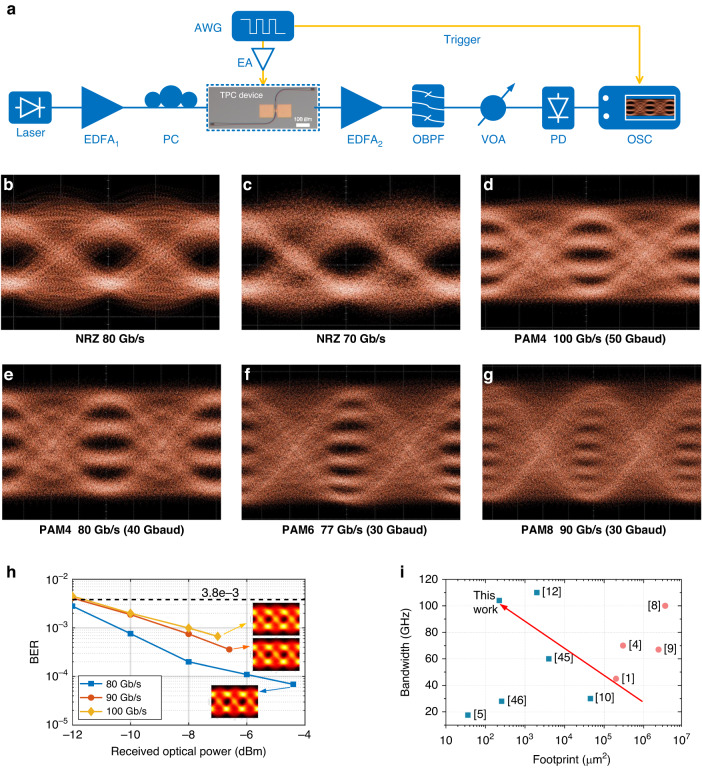
Data modulation testing. **a** Measurement setup for data transmission testing. Eye diagrams for the NRZ signals at data rates of **b** 80 Gb/s and **c** 70 Gb/s, the PAM-4 signals at data rates of **d** 100 Gb/s and **e** 80 Gb/s, the PAM-6 signal at a data rate of **f** 77 Gb/s, and the PAM-8 signal at a data rate of **g** 90 Gb/s. **h** The BER versus ROP curves for different NRZ signals. Insets illustrate the calculated eye diagrams after DSP for the recovered 80, 90, and 100 Gb/s NRZ signals. **i** Measured footprint-bandwidth performance comparison of various reported thin film LN modulators. The circle symbols correspond to the results of MZI modulators, and the rectangle symbols correspond to those of cavity-based modulators. OSC oscilloscope

We use the large bandwidth and ultracompact topological modulator to generate advanced modulation formats of up to 100 Gb/s. The experimental setup is shown in Fig. 4a (see “Methods” for the details of the setup). Figure 4b, c illustrates the raw optical eye diagrams for the NRZ signals at different bit rates of 80 and 70 Gb/s with signal-to-noise ratios (SNRs) of 3.43 and 3.62, respectively; clear open eyes are obtained. Moreover, the received optical power (ROP) sensitivities of 80, 90, and 100 Gbaud NRZ signals are investigated. In the testing, the digital storage oscilloscope was utilized to capture the photocurrent after the photodiode (PD), and the signal was processed with offline digital signal processing (DSP), which can be found in Supplementary Note S8. The bit error rate (BER) of the 80, 90, and 100 Gb/s NRZ signals are all below the 7% hard-decision forward error correction (FEC) threshold of 3.8 × 10^−3^ (Fig. 4h). The insets of Fig. 4h plot the calculated eye diagrams after DSP for the recovered 80, 90, and 100 Gb/s NRZ signals. The bit-switching energy is given by *CV*_pp_^2^/4 for the NRZ signals, which is ~5.4 fJ/bit for our topological modulator. The ultralow energy consumption is attributed to the small topological cavity and the low capacitance.

Four-level pulse amplitude modulation (PAM-4), PAM-6, and PAM-8 modulation formats at high baud rates are utilized to further increase the data rates supported by the topological modulators. Figure 4d–g show the clear open raw optical eye diagrams for the PAM-4 signals at rates of 100 Gb/s (50 Gbaud) and 80 Gb/s (40 Gbaud), the PAM-6 signal at a rate of 77 Gb/s (30 Gbaud) and the PAM-8 signal at a rate of 90 Gb/s (30 Gbaud).

The demonstrated topological modulator shows excellent performance in terms of device footprint and modulation bandwidth (Fig. 4i). In Table 1, we compare the performance of our topological modulator with state-of-the-art thin-film LN modulators based on MZI and cavity structures. Our demonstrated topological modulator exhibits an ultracompact footprint of 1.6 × 140 μm^2^, which is one to four orders of magnitude less than reported thin film LN modulators with bandwidths over 28 GHz. Additionally, our modulator features the advantages of large bandwidth and low power consumption, enabling the generation of 100 Gb/s NRZ and 100 Gb/s PAM-4 signals.

**Table 1:** Performances of various state-of-the-art thin film LN modulators

Structures	Footprint	*V*_π_*L*/tuning efficiency	Extinction ratio	Bandwidth	*V*_pp_	Data rate	Energy consumption
LN MZI on silicon^1^	100 × 20000 μm^2^	2.8 Vcm	30 dB	45 GHz	0.2 V	70 Gb/s	/
Hybrid LN-silicon MZI^4^	~100 × 3000 μm^2^	2.2 Vcm	40 dB	>70 GHz	4 V	100 Gb/s OOK	170 fJ/bit
LN MZI on quartz^8^	175 × 20,000 μm^2^	2.6 Vcm	20 dB	>100 GHz	/	/	/
Ring-assisted MZI^9^	700 × 3400 μm^2^	0.35 Vcm	20 dB	>67 GHz	0.75 V	224 Gb/s PAM-4	2.7 fJ/bit
Racetrack^10^	100 × 450 μm^2^	7 pm/V	6.5 dB	30 GHz	5.66 V	40 Gb/s NRZ	240 fJ/bit
Racetrack SiN loaded LN^11^	600 × 210 μm^2^	/	9 dB	/	/	70 Gb/s NRZ	212 fJ/bit
Bragg grating^45^	10 × 400 μm^2^	/	53.8 dB	60 GHz	0.9 V	100 Gb/s NRZ	/
BIC photonic crystal^46^	2.1 × 123 μm^2^	1.5 pm/V	/	28 GHz	/	/	/
FP cavity^12^	4 × 500 μm^2^	7 pm/V	20 dB	>110 GHz	2 V	100 Gb/s OOK	4.5 fJ/bit
Photonic crystal^5^	1.2 × 30 μm^2^	16 pm/V	11.5 dB	17.5 GHz	2 V	11 Gb/s NRZ	22 fJ/bit
Topological modulator (this work)	**1.6** **×** **140** **μm**^**2**^	11 pm/V	32 dB	**104** **GHz**	2 V	100 Gb/s NRZ 100 Gb/s PAM-4	5.4 fJ/bit

Bold values highlight the size and bandwidth of our topological modulator, which represents a significant improvement over previously reported results

## Discussion

We have demonstrated a topological interface state in a 1D microstructure lattice based on the classic SSH model using an integrated thin film LNOI platform. The LN-based interface state, which arises from band crossing, exhibits the advantages of a high Q factor, small mode volume, single mode operation, avoidance of mode number control, and robustness to defects or disorders. To the best of our knowledge, we have implemented the first high-speed topological electro-optic modulator using this topological boundary state. Owing to the strong optical confinement of the interface state, the size of the topological modulator is only 1.6 × 140 μm^2^. Due to good electro-optic overlap and peaking enhancement in the topological cavity, the LN-based interface state is capable of a large modulation bandwidth of 104 GHz. High-speed modulation of up to 100 Gbaud NRZ signal is achieved with switching energy as low as 5.4 fJ/bit, which is attributed to the small device footprint and short electrode length. Furthermore, a 100 Gb/s PAM-4 signal is enabled by the topological modulator.

Our topological modulator shows great promise for applications of high-speed modulation in fully integrated LN photonics, but there are some improvements needed for future work. First, the width and thickness of the microelectrodes can be further optimized to obtain a smaller RC time constant. Second, the unit cells of the TPC can be decreased to achieve a lower Q factor, thus reducing the limitation of the photon lifetime on the electro-optic bandwidth. Third, considering the wavelength drift of the topological interface states due to the fabrication or temperature variations in practical applications, a power-efficient thermo-tuning element can be integrated into the LN topological cavity with the capability of resonance tuning. Finally, due to its compact footprint, low switching energy, and the absence of complicated resonant mode control, a large number of LN topological cavities are attainable for integration on the same chip to achieve a communication link with an aggregate data rate beyond Tb/s using wavelength division multiplexing technology^43^. In addition to the topological boundary state-based microcavity modulator proposed in this work, topological slow light waveguides have higher group refractive indices and weaker backscattering, which are expected to be used for MZI electro-optic modulators to achieve shorter modulation arms and higher modulation efficiency. Two-dimensional and even higher-dimensional topological photonic crystal structures are also promising for achieving high-speed electro-optic modulators, which can bring advantages of anti-backscattering, unidirectional propagation, robustness, and immunity to disorder. However, there are also challenges to overcome. For instance, the relatively complex nature of two-dimensional topological optical structures presents difficulties in designing efficient electric field structures, potentially reducing the electro-optic overlap efficiency.

## Materials and methods

### Device fabrication

The fabrication process starts from an X-cut LNOI wafer with a 300-nm-thick LN layer and a 2-μm-thick buried silica layer (purchased from NanoLN). A 300-nm-thick silicon-nitride layer is deposited on the LNOI substrate using plasma-enhanced chemical vapor deposition (PECVD). The topological structures and grating couplers are patterned on the resist (AR-P 6200.09) and transferred to the silicon-nitride layer by electron-beam lithography (EBL, Vistec EBPG 5200^+^) and inductively coupled plasma (ICP) dry etching (NMC), respectively. After residue removal, the microelectrodes and contact pads (10 nm Ti/300 nm Au) are deposited by electron-beam evaporation and patterned by the lift-off process.

### Numerical simulation

The band diagrams of the topological photonic crystal are calculated by the finite element method. The equivalent refractive index of the waveguides with subwavelength-scale rectangular air holes is calculated using effective-medium theory (see Supplementary Note S2). The transmission spectrum, Q factor, and mode volume of the topological cavity are simulated by the finite-difference time-domain (FDTD, Lumerical FDTD solutions) method with perfectly matched layer boundary conditions.

### Optical characterization

The topological devices are characterized by using a tunable laser scanning system (EXFO T100S-HP-CLU-M-CTP10-00). On-chip grating couplers are used to couple light into/out of the silicon-nitride-loaded LN waveguides (see Supplementary Note S9). We measured the electro-optic tuning of the boundary states at different voltages using a voltage-current source meter (Keithley 2400).

### Setup for sideband testing

The light is coupled into the topological device using grating couplers. As shown in Fig. 3a, the RF signal generated by an arbitrary waveform generator (AWG, Keysight M8195A) is applied to the microelectrodes of the topological device by a ground-signal (GS) probe with an operating bandwidth of 40 GHz (InfinityQuad Probe). The modulated optical signal is recorded for each RF frequency by an optical spectrum analyzer (Yokogawa AQ6370B).

### Setup for high-speed measurements

The small-signal response measurements are performed using a 110-GHz LCA and a 90-GHz photodiode (XPDV4120R-WFFP). For the raw eye diagram measurements, an AWG with a sampling rate of 120 GS/s (Keysight M8194) and an RF amplifier (SHF S807C, 3-dB bandwidth: 55 GHz) are utilized to generate a pseudorandom bit sequence (PRBS), and the signals are connected to the LN topological modulator by a high-bandwidth GS probe with a driving peak-to-peak voltage of ~2 V. Finally, the modulated light is recorded by an electrical sampling oscilloscope (Keysight N1092). For the ROP sensitivity testing, the details for the experiment and transceiver DSP flow charts are provided in Supplementary Note S8.

### Supplementary information

Supplementary Information
