# Supplementary material for: High-speed electro-optic modulation in topological interface states of a one-dimensional lattice
Source: Light Sci Appl. 2023 Aug 29;12:206. doi: 10.1038/s41377-023-01251-x (PMC10465510; doi:10.1038/s41377-023-01251-x)
Supplement: Supplementary file 1 — Supplementary Information [file 41377_2023_1251_MOESM1_ESM.docx]

**Supplementary Information for**

**High-speed electro-optic modulation in topological interface states of a one-dimensional lattice**

**Yong Zhang^1*^, Jian Shen^1^, Jingchi Li^1^, Hongwei Wang^1^, Chenglong Feng^1^, Lei Zhang^1^, Lu Sun^1^, Jian Xu^2^, Ming Liu^2^, Ying Wang^2^, Yonghui Tian^3^, Jianwen Dong^4^, and Yikai Su^1*^**

1. State Key Laboratory of Advanced Optical Communication Systems and Networks, Department of Electronic Engineering, Shanghai Jiao Tong University, Shanghai 200240, China

2. Center for Advanced Electronic Materials and Devices, Shanghai Jiao Tong University, Shanghai 200240, China

3. Institute of Microelectronics and Key Laboratory for Magnetism and Magnetic Materials of MOE, School of Physical Science and Technology, Lanzhou University, Lanzhou 730000, Gansu, China

4. State Key Laboratory of Optoelectronic Materials and Technologies & School of Physics, Sun Yat-sen University, Guangzhou 510275, China

* Correspondence: Yong Zhang (yongzhang@sjtu.edu.cn)

Department of Electronic Engineering

Shanghai Jiao Tong University

Shanghai 200240, China

Email: yongzhang@sjtu.edu.cn

* Correspondence: Yikai Su (yikaisu@sjtu.edu.cn)

Department of Electronic Engineering

Shanghai Jiao Tong University

Shanghai 200240, China

Email: yikaisu@sjtu.edu.cn

Supplementary Note S1: Zak phase and symmetry property of the interface state


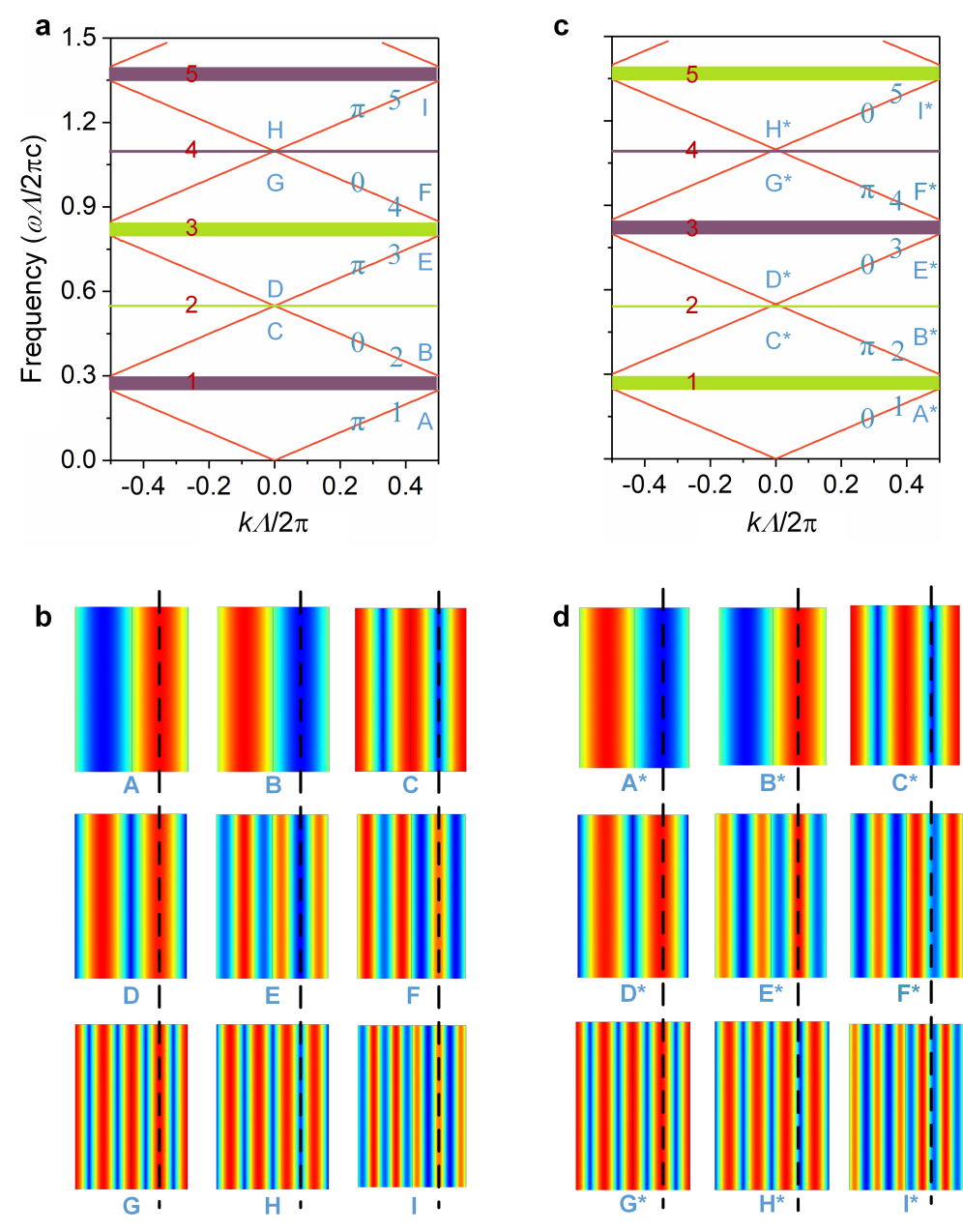


Fig. S1. Bulk band diagram and electric field distributions of the TPCs. Band structures of the (a) left and (c) right TPC with parameters of *ε*_a_ = 3.39, *ε*_b_ = 3.24, *μ*_a_ = *μ*_b_ = 1. Simulated electric field distributions of the band-edge states (points A - I and A* - I*) for the (b) left and (d) right TPC. The black dashed lines indicate the origin of our discussion (the center of layer A).

We discuss the relationship between the Zak phases in each isolated band and the symmetry properties of the interface states at the two symmetry points of the Brillouin region in this section. **Fig. S1**a and S1c show the band dispersion of the left and right topological photonic crystals (TPCs) based on dielectric AB layered structures, respectively. As an example, let us consider the 2nd bands of the two TPCs firstly (marked by blue numbers), in which the Zak phases change by π for the two TPCs. The electric distributions *E_n,k_*(*y,z*) of the interface states at the symmetry points B, C, B*, and C* of the left and right TPCs are depicted in **Fig. S1**b and S1d. The center of layer A is selected to be the origin of our discussion, as shown in the black dashed lines. For 1D photonic systems with inversion symmetry, the Zak phase of the *n*th band is zero if either |*E_n,k=0_*(*y=*0*,z*)| = |*E_n,k=π/Λ_*(*y=*0*,z*)| = 0 or |*E_n,k=0_*(*y=*0*,z*)| ≠ 0; |*E_n,k=π/Λ_*(*y=*0*,z*)| ≠ 0. Otherwise, the Zak phase is π ^1^. The Zak phase of the 2nd band of the left TPC is zero since the amplitude of the electric field (|*E_n,k=0_*(*y=*0*,z*)| and |*E_n,k=π/Λ_*(*y=*0*,z*)|) of the points B and C are both zero at the origin. For the right TPC, the amplitude of the electric field of point C* is zero, while that of point B* is at maximum. Thus, the Zak phase of the 2nd band of the right TPC changes to π. Depending on the electric field intensity distribution at these points of D and E, F and G, H and I, B* and C*, D* and E*, F* and G*, and H* and I*, we can obtain the Zak phase of each energy band, respectively, as depicted by blue letters in **Fig. S1**a and S1c.

Band crossing and phase transition can be confirmed by the changes in the symmetries of the interface states. As an example, let us consider the 1st band gaps of the two TPCs firstly (marked by red numbers), in which the gap topological invariants vary for the two TPCs. The amplitude of the electric field of points B and A* are both zero at the origin, while that of points A and B* are both at maximum. However, the amplitude at points C and C* are almost the same and not dependent on the band inversion. It is exactly the switching of the symmetry of the two edge states at the 1st gap that causes the distinct Zak phases of the 1st and 2nd bands in the left and right TPCs. A similar edge-state symmetry transformation is observed in the 3rd and 5th band gaps, while it is not found in band gaps 2 and 4. This method can be used to predict the presence or absence of topological bound states in each band gap.

Supplementary Note S2: Effective-medium theory to bridge integrated waveguide with air holes and dielectric AB layered structure


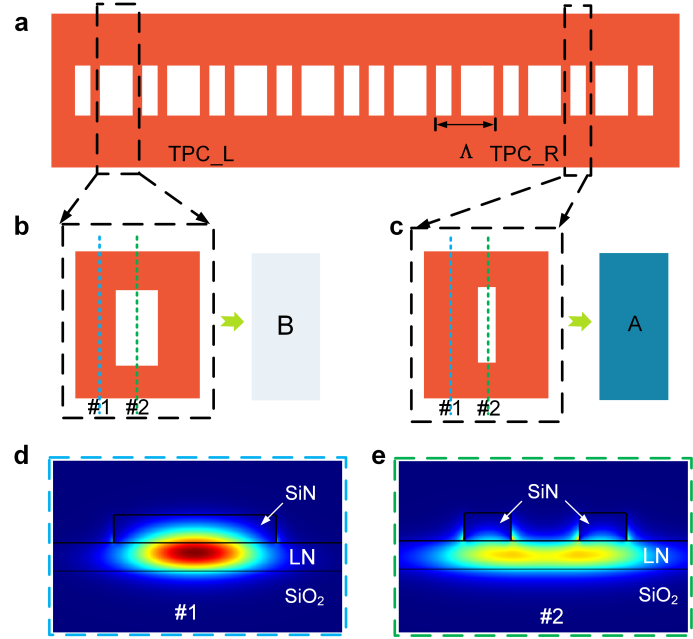


**Fig. S2. Effective medium theory for integrated periodic topological structure.** (a) Schematic configuration of the integrated periodic topological structure. (b) and (c) zoomed-in view of half a unit cell. Calculated mode distributions at positions (d) #1 and (e) #2.

For the on-chip device design, periodic rectangular air holes are utilized to tailor the effective index of integrated waveguides and replace the dielectric AB layered structures in the TPC cavity, as depicted in **Fig. S2**a. **Fig. S2**b-c shows a zoomed-in view of half a unit cell. The effective refractive index of half a unit cell is calculated by using the effective-medium theory. For a conventional one-dimensional periodic structure with a period much smaller than the wavelength of the light, the guiding wave is confined in the subwavelength structure rather than being diffracted or scattered. Half a unit cell with a rectangular air hole acts as a homogeneous medium with an effective refractive index of:

$$n_{eq}=(1-\eta)n_{eff1}+\eta n_{eff2}$$

where *η* is the duty cycle of the rectangular air hole in half a unit cell, and *n_eff_*_1_ and *n_eff_*_2_ are the effective indices at positions #1 and #2, respectively. The simulated mode distributions at positions #1 and #2 are plotted in **Fig. S2**b-c, respectively. The corresponding effective indices *n_eff_*_1_ and *n_eff_*_2_ can be calculated using the finite-difference eigenmode (FDE) method.

Supplementary Note S3: Simulated results of the topological cavity

The FDTD simulations employ 170 unit cells, with perfectly matched layer (PML) boundary conditions. The topological structure is excited by the fundamental TE mode supported by the hybrid Si_3_N_4_-LN waveguide. The simulated optical field distribution of the Si_3_N_4_-LN waveguide and the topological boundary state is monitored at the Thru port of the topological structure and the center of the LN thin film, respectively.

The extinction ratio is an important metric to evaluate the LN-based integrated topological cavity. We calculate the extinction ratios of the topological edge modes for different D1 and D2 values (**Fig. S3**). The simulated extinction ratio of the topological cavity can reach higher than 33 dB.


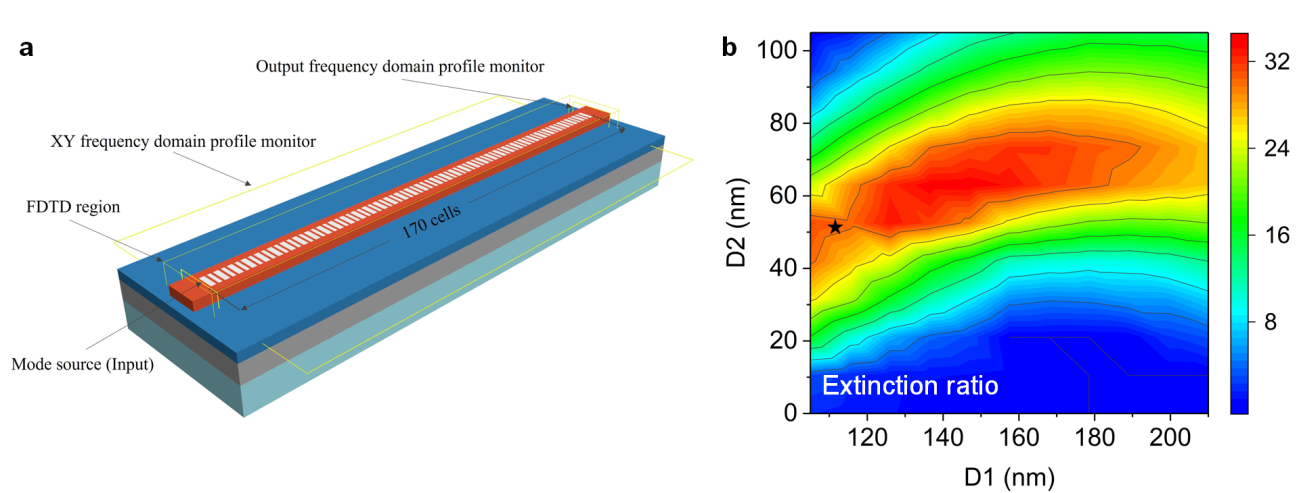


**Fig. S3.** (a) Schematic of the simulated topological cavity structure in the FDTD simulation. (b) Simulated extinction ratio of the topological cavity.

To verify the robustness of the topological photonic crystal microcavity to fabrication errors, we conduct additional simulations. We vary the widths D1 and D2 of the middle 10 unit cells in the topological cavity (as shown in the inset of **Fig. S**4a). It can be observed that as the width decreases by 20nm or increases by 20nm, the topological edge states persist, with only minor changes in their Q-factor and mode volume. The insertion loss and extinction ratio of the topological edge states remain nearly unchanged, while the wavelength position shifts towards shorter wavelengths. Furthermore, we alter the positions of the middle 10 unit cells in the topological microcavity (as depicted in the inset of **Fig. S**4c). The simulations reveal that even when these 10 units are shifted downwards by 30nm or upwards by 30nm, the topological edge states continue to exist with virtually no changes in their Q-factor, mode volume, insertion loss, extinction ratio, and resonant wavelength position. These findings demonstrate the excellent robustness of the proposed one-dimensional Si_3_N_4_-LN topological photonic crystal microcavity.


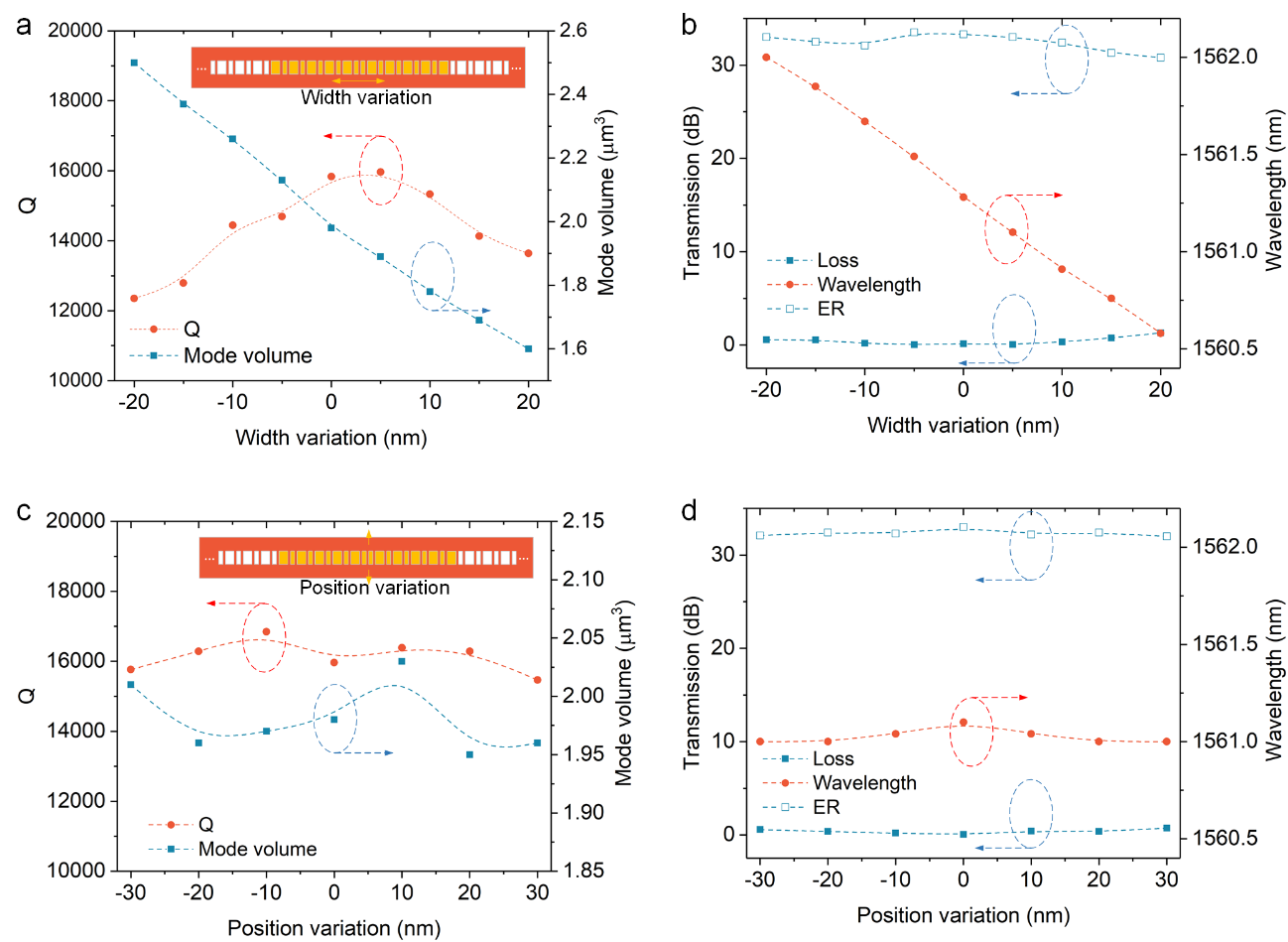


**Fig. S4.** **Simulation results of the topological cavity with different defects.** (a) Simulated Q factor and mode volume, (b) Simulated loss, extinction ratio, and wavelength of the cavities with defects obtained by increasing or decreasing the air hole widths in the yellow region. (c) Simulated Q factor and mode volume, (d) Simulated loss, extinction ratio, and wavelength of the cavities with defects obtained by altering the positions of the air holes in the yellow region.

Supplementary Note S4: Mode field analysis of the Zak phase in the integrated topological interface states


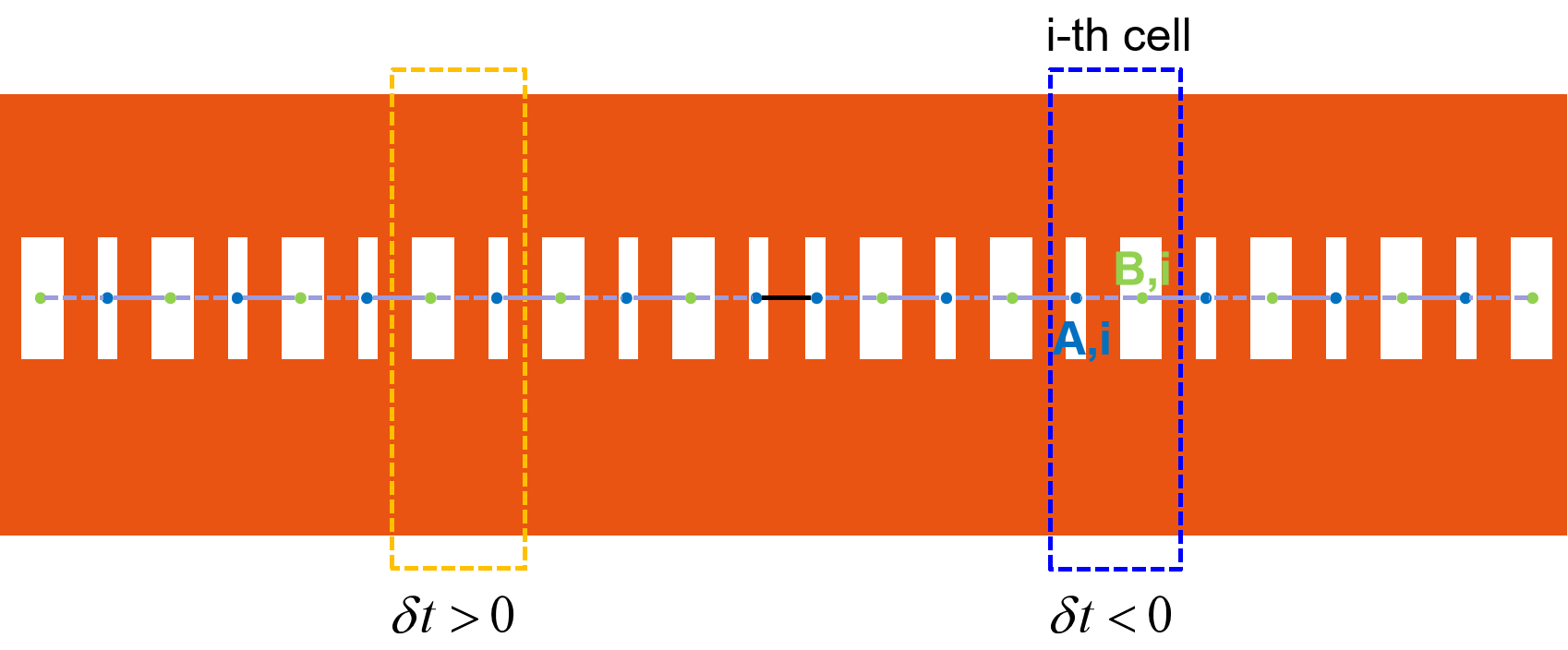


**Fig. S5.** **Su-Schrieffer-Heeger theoretical model of the one-dimensional Zak phase.**

Using the tight-binding approximation model, we can obtain the Hamiltonian for the Zak phase system (TPC_L or TPC_R) as follows ^2^:

$$H=\sum_{n=1}^{N} (t+\delta t)c_{A,n}^{\dagger}c_{B,n}+\sum_{n=1}^{N-1} (t-\delta t)c_{A,n+1}^{\dagger}c_{B,n}+h.c.$$

where $c_{A(B),n}^{\dagger}$ and $c_{A(B),n}$ are the creation and annihilation operators on A (or B) sublattice site (A(B), n) in the *n*th unit cell, the hopping amplitude in the unit cell is defined as *t* + *δt* and *t* – *δt*, respectively. To diagonalize the Hamiltonian, performing the Fourier transformation:

$$a_{k}=\frac{1}{\sqrt{N}}\sum_{n} e^{-ik\cdot n\Lambda}c_{A,n}$$

$$b_{k}=\frac{1}{\sqrt{N}}\sum_{n} e^{-ik\cdot n\Lambda}c_{B,n}$$

where *N* is the number of unit cells. Thus the Hamiltonian is:

$$H=(t+\delta t)\sum_{k\in(-\pi/\Lambda,\pi/\Lambda)} (a_{k}^{\dagger}b_{k}+b_{k}^{\dagger}a_{k})+(t-\delta t)\sum_{k\in(-\pi/\Lambda,\pi/\Lambda)} (e^{ik\Lambda}a_{k}^{\dagger}b_{k}+e^{-ik\Lambda}b_{k}^{\dagger}a_{k})$$

Introducing the spinor $\psi_{k}=(a_{k},b_{k})^{T}$ into Eq. (5), the Hamiltonian can be reduced to:

$$H=\sum_{k\in(-\pi/\Lambda,\pi/\Lambda)} \psi_{k}^{\dagger}\{[(t+\delta t)+(t-\delta t)\cos k\Lambda]\sigma_{x}+(t-\delta t)\sin k\Lambda\sigma_{y}\}\psi_{k}$$

when *k* = *π*/Λ, introducing the transformation, $\sigma_{x}\to\sigma_{z}$, $\sigma_{y}\to\sigma_{x}$and $k\to\delta k+\pi/\Lambda$, the Hamiltonian is simplified as:

$$H=-(t-\delta t)\delta k\Lambda\sigma_{x}+2\delta t\sigma_{z}$$

We can know the basic structure of the one-dimensional Zak phase to obtain its Dirac equation:

$$H=-i(t-\delta t)\Lambda\partial_{x}\sigma_{x}+m(x)\sigma_{z}$$

where $m(x)=\left\{\begin{matrix} 2\delta t, x>0 \\ 0, x=0 \\ -2\delta t, x<0 \end{matrix}\right.$, $\delta t<0$. Thus, we can obtain the following relation for the expression of its general solution:

$$|\psi\rangle\propto\frac{1}{\sqrt{2}}\left(\begin{matrix} 1 \\ i \end{matrix}\right)e^{-\left|\frac{2\delta t}{t-\delta t}\cdot\frac{x}{\Lambda}\right|}$$

Using Eq. (9), we can theoretically calculate the decay curves of the electric field amplitude in the TPC structure (**Fig. S6**). *δt* is fitted to be -0.0042*t* for the right TPC with D1 = 150 nm, D2 = 80 nm, and D3 = 674 nm. The theoretically calculated decay curves coincide with the electric field envelope curves from the FDTD simulation (**Fig. S6**).


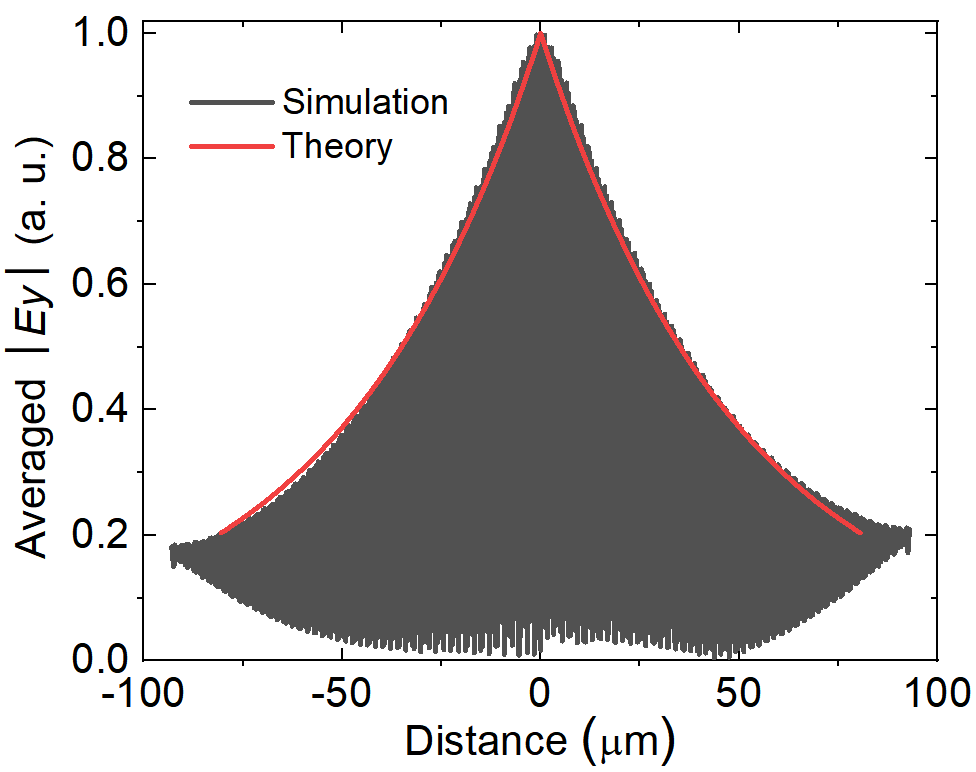


**Fig. S6. Theoretically calculated decay curves of the electric field amplitude and the electric field envelope curves from the FDTD simulation.**

Supplementary Note S5: Electro-optic simulation of the topological cavity with gold electrodes

The electric field is applied to the topological cavity through planar microelectrodes beside the waveguide. The simulated static electric field distribution is plotted in **Fig. S7**a when a bias voltage of 1 V is applied to the electrodes. The overlap between the optical and electrical fields is important to increase the modulation efficiency, which can be quantified by the electro-optic integral. The electro-optic integral Γ is ^3^

$$\Gamma=\frac{g}{V}\frac{\iint E_{x,op}^{2}E_{e}dxdz}{\iint E_{x,op}^{2}dxdz}$$

where *g* is the electrode separation, *V* is the voltage on the electrodes, *E_x,op_* is the optical field, and *E_e_* is the electrical field. **Fig. S7**b plots simulated Γ as a function of the Si_3_N_4_ thicknesses for different LN thicknesses. It indicates that electro-optic integral Γ increases as the LN thickness increases and the Si_3_N_4_ thickness decreases.


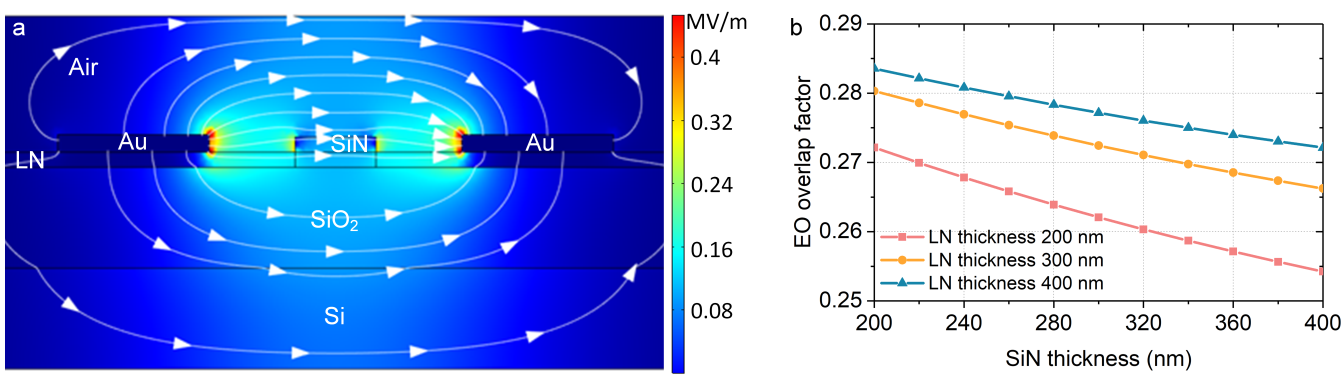


**Fig. S7. Electro-optic simulation of the topological modulator.** (a) Simulated static electric field distribution of the Si_3_N_4_-LN hybrid waveguide induced by the planar electrodes. The applied voltage is 1 V. (b) Numerically calculated electro-optic overlap integral Γ as a function of LN and Si_3_N_4_ thicknesses.

The capacitance of the micro-electrodes is calculated using the finite element method. Zero-charge boundary conditions are applied to the outer edges of the simulation cell. The permittivity of the materials used in the model is presented in **Table S1**. The width, thickness, and gap of the micro-electrodes are 3 μm, 0.35 μm, and 5.5 μm, respectively. The simulated capacitance of the micro-electrodes as a function of the electrode length is plotted in **Fig. S8**. The capacitance per unit length is calculated to be 36 fF mm^-1^. The calculated capacitance of the topological modulator with an electrode length of 150 μm is 5.4 fF.

**Table S1**. The permittivity of the materials used in the model.

| Material | Permittivity |
| --- | --- |
| Silicon nitride | 9.7 |
| Lithium niobate | 28 |
| SiO_2_ | 3.9 |





**Fig. S8. Capacitance of the micro-electrodes as a function of the length.** The line corresponds to a linear fit of the calculated capacitance.

Supplementary Note S6: RC-limited bandwidth of the lumped-element topological modulator

The electrode of the topological cavity is lumped type, thus acts like a capacitor with its bandwidth limited by the product of capacitance, *C*, and load resistance, *R*. We can calculate the RC-limited bandwidth *f_BW_* by using

$$f_{BW}=\frac{1}{\pi RC_{0}L}$$

where *C*_0_ is the capacitance per unit length and *L* is the electrode length.

For our topological modulator with an electrode length of 150 µm, the RC-limited bandwidth exceeds 1.1 THz (**Fig. S9**). This is attributed to the short electrode length and small capacitance per unit length.


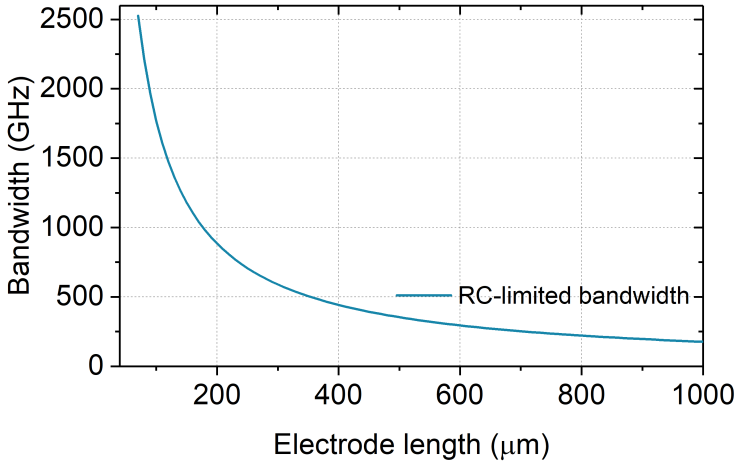


**Fig. S9. Calculated RC-limited bandwidth for different electrode lengths.**

Supplementary Note S7: Perturbative derivation of S_21_ response of the topological cavity modulation


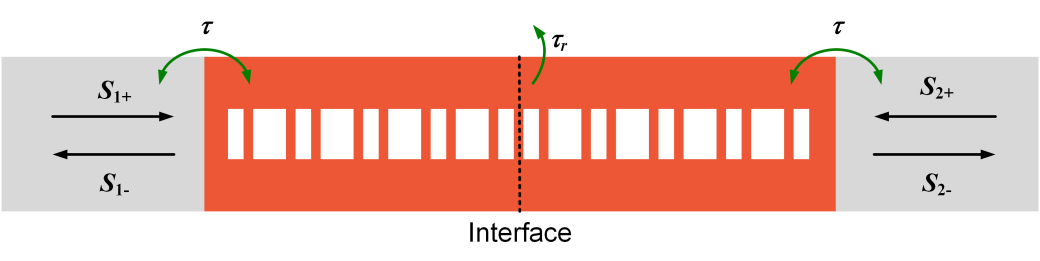


**Fig. S10. Schematic illustration of a Zak-phase-based resonator.**

As shown in **Fig. S10**, when the light is incident from the left side with $s_{2+}=0$, the transmission equation becomes:

$$\frac{da}{dt}=-i\omega_{r}a-\frac{a}{\tau_{r}}-\frac{2a}{\tau}+\sqrt{\frac{2}{\tau}}s_{1+}$$

$$s_{1-}=-s_{1+}+\sqrt{\frac{2}{\tau}}a$$

$$s_{2-}=\sqrt{\frac{2}{\tau}}a$$

where *τ* denotes the coupling between the topological cavity and the input/output waveguide, and *τ_r_* is the radiation coupling between the topological cavity and the cladding. Assuming that the applied voltage induces a small index change and a small perturbation in the topological cavity response, the perturbation for the amplitude *a* inside the topological cavity and the resonant frequency *ω_r_* is *δa* and *δω_r_*, respectively. Eq. (12) is transformed into:

$$\frac{\partial a}{\partial t}+\frac{\partial\delta a}{\partial t}=-i(\omega_{r}+\delta\omega_{r})(a+\delta a)-(a+\delta a)/\tau_{r}-\frac{2(a+\delta a)}{\tau}+\sqrt{\frac{2}{\tau}}s_{1+}$$

Disregarding the second-order approximation term and subtracting from Eq. (12), Eq. (15) is rewritten as:

$$\frac{\partial\delta a}{\partial t}=-(i\omega_{r}+\frac{2}{\tau}+\frac{1}{\tau_{r}})\delta a-ia\delta\omega_{r}$$

Both sides of the equal sign of Eq. (16) are simultaneously multiplied by $e^{(i\omega_{r}+\frac{2}{\tau}+\frac{1}{\tau_{r}})t}$, and $\delta a=\overline{\delta a}e^{-(i\omega_{r}+\frac{2}{\tau}+\frac{1}{\tau_{r}})t}$, then Eq. (16) can be reduced to:

$$\frac{\partial\overline{\delta a}}{\partial t}=-\overline{\delta a}/\tau_{r}-ia\delta\omega_{r}e^{(i\omega_{r}+\frac{2}{\tau}+\frac{1}{\tau_{r}})t}$$

Using $a=\bar{a}e^{-i\omega_{0}t}$, $s_{2-}=\overline{s_{2-}}e^{-i\omega_{0}t}$, $s_{1+}=\overline{s_{1+}}e^{-i\omega_{0}t}$ and assuming the time varying resonant frequency $\delta\omega_{r}=\overline{\delta\omega_{r}}\cos(\omega_{m}t)=\overline{\delta\omega_{r}}(e^{i\omega_{m}t}+e^{-i\omega_{m}t})$, where *ω_m_* and *ω*_0_ represent the modulation frequency and the input light frequency, respectively, Eq. (17) is rewritten as:

$$\frac{\partial\overline{\delta a}}{\partial t}=-i\frac{1}{2}\overline{a}\overline{\delta\omega_{r}}(e^{(i\omega_{r}-i\omega_{0}+i\omega_{m}+\frac{2}{\tau}+\frac{1}{\tau_{r}})t}+e^{(i\omega_{r}-i\omega_{0}-i\omega_{m}+\frac{2}{\tau}+\frac{1}{\tau_{r}})t})$$

Then we can obtain *δa* to be:

$$\delta a=-i\frac{1}{2}\overline{a}\overline{\delta\omega_{r}}(\frac{1}{i\omega_{r}-i\omega_{0}+i\omega_{m}+\frac{2}{\tau}+\frac{1}{\tau_{r}}}e^{(-i\omega_{0}+i\omega_{m})t}+\frac{1}{i\omega_{r}-i\omega_{0}-i\omega_{m}+\frac{2}{\tau}+\frac{1}{\tau_{r}}}e^{(-i\omega_{0}-i\omega_{m})t})$$

and the transmission $\overline{s_{2-}}$ to be:

$$\overline{s_{2-}}=\sqrt{\frac{2}{\tau}}\overline{a}-i\sqrt{\frac{1}{2\tau}}\overline{a}\overline{\delta\omega_{r}}(\frac{1}{i\omega_{r}-i\omega_{0}+i\omega_{m}+\frac{2}{\tau}+\frac{1}{\tau_{r}}}e^{i\omega_{m}t}+\frac{1}{i\omega_{r}-i\omega_{0}-i\omega_{m}+\frac{2}{\tau}+\frac{1}{\tau_{r}}}e^{-i\omega_{m}t})$$

We can derive the small signal response of the topological modulator to be:

$$S_{21,EO}=\left|\overline{s_{2-}}\right|^{2}-\left\langle\left|\overline{s_{2-}}\right|^{2}\right\rangle=\sqrt{\frac{2}{\tau}}\mathrm{Re}([\frac{\frac{1}{\tau}\overline{a}(-i\overline{a}\overline{\delta\omega_{r}})^{*}}{i\omega_{r}-i\omega_{0}+i\omega_{m}+\frac{2}{\tau}+\frac{1}{\tau_{r}}}+\frac{-i\frac{1}{\tau}\overline{a}\overline{\delta\omega_{r}}(\overline{a})^{*}}{i\omega_{r}-i\omega_{0}-i\omega_{m}+\frac{2}{\tau}+\frac{1}{\tau_{r}}}]e^{-i\omega_{m}t})$$

Assuming $\Delta\omega=\omega_{r}-\omega_{0}$, using $a=\bar{a}e^{-i\omega_{0}t}$,$s_{1+}=\overline{s_{1+}}e^{-i\omega_{0}t}$ and Eq. (12), we can obtain:

$$\bar{a}=\frac{\sqrt{\frac{2}{\tau}}\overline{s_{1+}}}{i\Delta\omega+\frac{2}{\tau}+\frac{1}{\tau_{r}}}$$

Using Eqs. (21) and (22), the small-signal response of the topological cavity modulation can be calculated (**Fig. S11**). The wavelength detuning is defined as the difference between the input optical wavelength *λ*_0_ and the resonance *λ_r_* of the interface state. The modulation frequency of the peak in the S_21_ response corresponds approximately to the detuning between the input optical frequency *ω*_0_ and the resonant frequency *ω_r_*. The wavelength detuning of 0.2, 0.3, 0.4, and 0.5 nm correspond to the detuning points of 3, 4.5, 6.5, and 8 dB down from the on-resonance transmission maximum power level, respectively. The calculated 3-dB electro-optic bandwidth of the topological modulator is 47, 67, 88, and 109 GHz at wavelength detuning points of -3, -4.5, -6.5, and -8 dB, respectively. The simulated bandwidth closely matches the experimentally tested bandwidth. In the high-frequency range, the experimental test curve exhibits a steeper decline, which can be attributed to the back reflections caused by the probe tips at the RF output port of the LN chip.


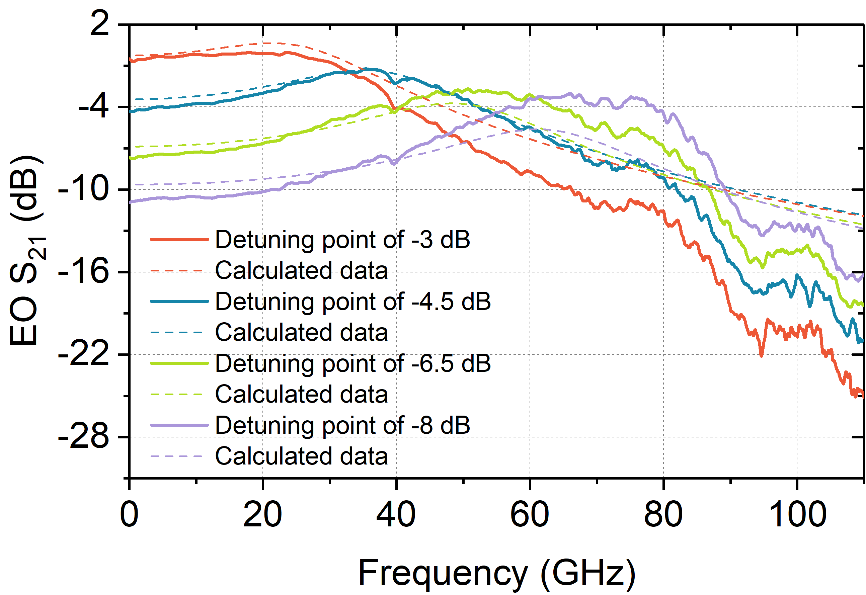


**Fig. S11. Calculated and measured EO small-signal response of the topological cavity modulation for different detuning wavelengths.**

Supplementary Note S8: Received optical power sensitivity testing

We carry out the high-speed transmission experiment with the topological modulator. A Nyquist NRZ signal is generated with a roll-off factor of 0.01. After resampling to 100 GSa s^-1^ to match the sampling rate of the digital-to-analog converter (DAC), the signal is sent to a 100 GSa s^-1^ DAC (Micram DAC4). The signal output from the DAC is amplified by a 23-dB electrical amplifier (EA) and inserted into the topological modulator with a high-speed probe and cable. In the experiment, the detuning wavelength was optimized for different signals to provide the best performance. A continuous-wave (CW) light from a laser is boosted with an erbium-doped fiber amplifier (EDFA) and then serves as the optical carrier for E/O conversion. At the receiver, a variable optical attenuator (VOA) is employed to adjust the received optical power (ROP). Then, the signal is detected using a 70-GHz photodiode (PD), followed by a 160 GSa s^-1^ digital storage oscilloscope (DSO) (LeCroy 59Zi-A) to capture the photocurrent. After resampling, matched filtering, and synchronization, the captured signal is processed with a 61-tap least mean square-based feedforward equalizer (FFE) for compensating the channel response. Since the FFE causes high-frequency noise enhancement in the bandwidth-limited system, a post filter (PF) is applied to suppress the amplified noise, followed by the maximum-likelihood sequence decision to eliminate the known inter-symbol interference (ISI) introduced by the PF. Finally, the bit error rate (BER) calculation is performed by error counting of ~2×10^5^ bits. The transceiver DSP flow charts are presented in **Fig. S12**.


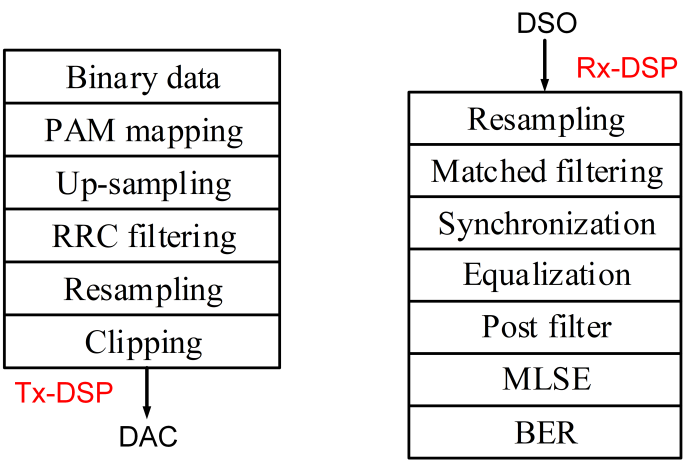


**Fig. S12. Transceiver DSP flow charts in the ROP sensitivity testing.**

Supplementary Note S9: Grating coupler for the fiber-to-chip coupling

Grating couplers are used to couple the light from a single-mode fiber into the Si_3_N_4_-LN hybrid waveguides. The period and the filling factor of the grating coupler are 1027 nm and 71%, respectively. **Fig. S13** shows the simulated mode profile and measured transmission spectrum of the grating coupler. The coupling loss and 3-dB bandwidth of the fabricated grating coupler are 6.8 dB port^-1^ and 53 nm, respectively. Apodized gratings are expected to match the diffracted wave to the mode profile of the fiber, leading to higher coupling efficiency.


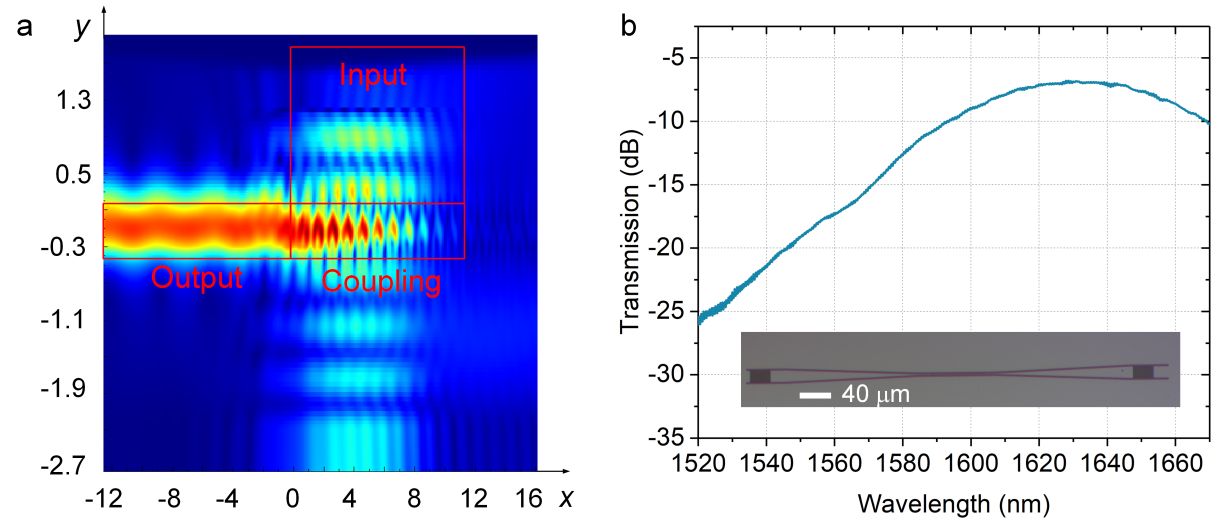


**Fig. S13. Grating coupler for** **Si_3_N_4_-LN hybrid chip.** (a) Simulated mode profile of the grating coupler. (b) Measured transmission spectrum of the fabricated grating coupler. Inset: optical microscope photo of the grating coupler.

**References:**

1. Zak, J. Berry's phase for energy bands in solids. *Physical Review Letters* **62**, 2747-2750 (1989).

2. Shen, S.-Q., *Topological Insulators* (Springer Berlin, Heidelberg, 2012).

3. Shen, J. et al. Ultralow-Power Piezo-Optomechanically Tuning on CMOS-Compatible Integrated Silicon-Hafnium-Oxide Platform. *Laser & Photonics Reviews* **16**, 2200248 (2022).
